# Improved production of recombinant human Fas ligand extracellular domain in *Pichia pastoris*: yield enhancement using disposable culture-bag and its application to site-specific chemical modifications

**DOI:** 10.1186/1472-6750-14-19

**Published:** 2014-03-11

**Authors:** Michiro Muraki

**Affiliations:** 1 Biomedical Research Institute, National Institute of Advanced Industrial Science and Technology (AIST), Central 6, 1-1-1, Higashi, Tsukuba, Ibaraki 305-8566, Japan

**Keywords:** Human Fas ligand, Extracellular domain, Production, *Pichia pastoris*, Yield enhancement, Site-specific chemical modification

## Abstract

**Background:**

A useful heterologous production system is required to obtain sufficient amounts of recombinant therapeutic proteins, which are often necessary for chemical characterization and engineering studies on the development of molecules with improved properties. Human Fas ligand extracellular domain (hFasLECD) is an agonistic death ligand protein that has potential applications for medical purposes. Site-specific chemical modifications can provide a powerful means for the development of engineered proteins with beneficial functions. This study aimed to enhance the yield of hFasLECD using a *Pichia pastoris* secretory expression system suitable for efficient production on a small laboratory scale, and further to provide procedures for its site-specific chemical modification without impairing the biological functions based on the developed production system.

**Results:**

A convenient cultivation system using a disposable plastic bag provided a three-fold increase in purification yield of tag-free hFasLECD as compared with the conventional system using a baffled glass flask. The system was further applied to the production of a mutant, which contains an additional reactive cysteine residue in the N-terminal tag-sequence region. Site-specific conjugations and cross-linking without impairing biological functions were achieved by reaction of the mutant hFasLECD with single maleimide group containing compounds and a linear polyethylene glycol derivative containing two maleimide groups at either end, respectively. All purified tag-free and chemically modified hFasLECDs showed an evident receptor binding activity in co-immunoprecipitation experiments mediated by wild-type and N-glycosylation site deficient mutant human Fas receptor extracellular domain derivatives. An *N*-Ethylmaleimide conjugated hFasLECD derivative demonstrated a significant cytotoxic activity against human HT-29 colorectal cancer cells.

**Conclusions:**

A new, efficient cultivation system for enhanced secretory production of hFasLECD using *P. pastoris* and an effective strategy for site-specific chemical modifications of hFasLECD were devised. The results obtained constitute the basis for biomedical applications including developments of novel therapeutic proteins and diagnostic tools targeted to related diseases and their biomarkers.

## Background

Abnormal apoptosis can cause many serious diseases, such as cancers and rheumatoid arthritis, in the human body [1]. Therefore, it has been assumed that the successful artificial control of apoptotic processes will play a substantial role in therapeutic interventions of these diseases [2,3]. Human Fas ligand is a major death ligand protein, which triggers the execution of cellular apoptosis via extrinsic pathway by specific binding of its extracellular domain to that of an agonistic molecule, human Fas receptor [4]. Heterologous expression is an important means to fulfill the requirements for obtaining sufficient amounts of therapeutic human proteins. Developing an efficient production system is often necessary not only for direct utilization as practical medicines, but also for chemical characterization and engineering studies [5,6]. Accordingly, functional recombinant wild-type human Fas ligand extracellular domain (hFasLECD) and its derivatives have been produced in heterologous systems using several kinds of expression hosts including *Escherichia coli*[7], *Pichia pastoris*[8-10] and *Dictyostelium discoideum*[7].

Chemical modification, represented by pegylation, is a powerful method for the development of engineered proteins with beneficial functions, which include prolonged therapeutic activity in circulating blood, by adding specific chemical properties into the target protein molecules [11]. It may be also useful for the structural characterization of interesting biological functions in native proteins. However, non-specific chemical modifications of protein molecules can interfere with the expression of their intrinsic therapeutic properties either by direct chemical transformation or by physical coverage of the critical functional groups. Site-specificity in the modification can contribute to the enhancement of useful functions and avoid unwanted effects of chemical modifications. In this connection, we have conducted several site-specific chemical modification studies with human lysozyme for the purpose of both the alteration of substrate specificity [12] and the clarification of the origin of carbohydrate recognition specificity [13].

The extracellular domain of human Fas ligand locates at the carboxyl-terminal region [amino acid residues (aa) 103-281] of whole molecule consisting of 281 aa, and independently exists as trimeric subunits without the help of other parts of the molecule under physiological conditions [4]. This domain contains three N-glycosylation sites (Asn 184, Asn 250 and Asn 260) and two cysteine residues (Cys 202 and Cys 233) forming a disulfide-bridge. In previous studies, we developed a secretory production system of recombinant hFasLECD using *P. pastoris* as the expression host, and reported that both the addition of N-terminal FLAG®-(Gly)_5_ tag sequence [9] and the deletion of the non-essential region in trimerization (aa 103-138) [10] significantly increased the secretion level of the products. We also showed that two asparagine residues (Asn 184 and Asn 250) could be mutated to glutamine residues without serious reduction of the secretion level [9], and that the remaining heterogeneous N-glycan chains attached to Asn 260 in the N-terminal FLAG®-(Gly)_5_ tagged double N-glycosylation sites mutant could be trimmed to homogeneous N-acetyl glucosamine residues without impairing the binding activity [10] toward a recombinant human Fas receptor extracellular domain (hFasRECD) derivative produced in silkworm larvae [14,15].

In this report, a marked increase in the production yield of tag-free hFasLECD achieved by the utilization of a disposable plastic bag as the cultivation vessel is described. This system was further applied to the secretory production of a mutant, which has an additional reactive cysteine residue within the above mentioned N-terminal FLAG®-(Gly)_5_ tag sequence. The details of site-specific chemical modifications of this mutant with maleimide group containing compounds as well as the characterizations of the purified reaction products concerning binding activity toward hFasRECD and cytotoxic activity against a cancer cell line will be presented.

## Results

### Enhanced yield of tag-free hFasLECD using disposable culture-bag

**Figure 1:**
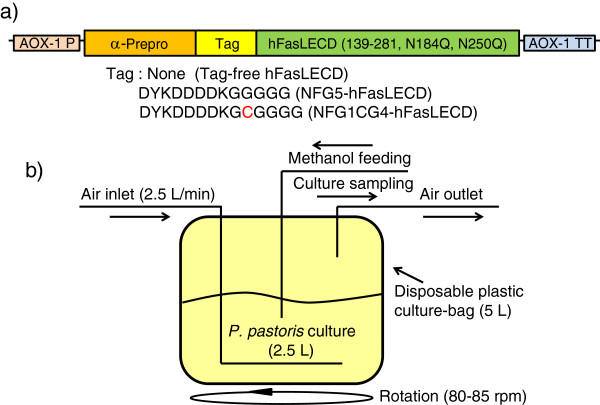
**Production of recombinant hFasLECDs in this study. a)** Gene structure of expression unit and variations in N-terminal tag sequences. AOX-1 P, *P. pastoris* alcohol oxidase 1 promoter region; α-Prepro, *Saccharomyces cerevisiae* α-factor secretion-signal sequence; Tag, tag sequence; hFasLECD (139-281, N184Q, N250Q), human Fas ligand extracellular domain containing deletion mutation from residue 103 to 138 and double substitution mutations (N184Q and N250Q); AOX-1 TT, *P. pastoris* alcohol oxidase 1 transcription termination region. **b)** Schematic presentation of *P. pastoris* cultivation system using disposable plastic bag.

**Figure 2:**
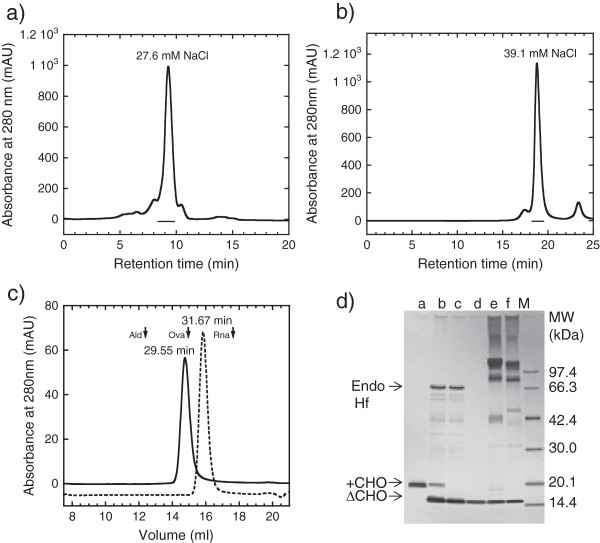
**Purification of N-glycan untrimmed and trimmed tag-free hFasLECDs. a)** Elution profile of N-glycan untrimmed tag-free hFasLECD sample (pre-purified by 1st Hi-Trap S cation-exchange chromatography) in 2nd cation-exchange chromatography. Used column: Resource S 6 ml. The region shown in underbar was collected and used for characterization in **c)**. NaCl concentration under principal peak eluting condition is described. **b)** Elution profile of N-glycan trimmed tag-free hFasLECD sample in 3rd cation-exchange chromatography. Used column: Mono S 1 ml. The region shown in underbar was collected and used for characterization in **c)**. NaCl concentration under principal peak eluting condition is described. **c)** Elution profiles of fractionated products in size-exclusion chromatography. Solid line: N-glycan untrimmed tag-free hFasLECD. Dashed line: N-glycan trimmed tag-free hFasLECD. Used column: Superdex 200 10/300 GL. Elution buffer: 50 mM sodium acetate plus 150 mM NaCl (pH 5.6). Flow rate: 0.5 ml/min. The peak retention time of each sample is described. Vertical arrows indicate the elution positions of molecular-weight size-markers [Ald, aldolase (158 kDa); Ova, ovalbumin (44 kDa); Rna, ribonuclease A (13.7 kDa) under the same conditions. **d)** SDS-PAGE analysis of purification course during N-glycan trimming with Endo Hf glycosidase and receptor-mediated co-immunoprecipitation using wild type and mutant hFasRECD-T-Fcs. Lanes: a, N-glycan untrimmed tag-free hFasLECD; b, after Endo Hf digestion; c, after Con A column fractionation; d, after Mono S column fractionation; e, co-immunoprecipitated materials using wild-type hFasRECD-T-Fc [15]; f, co-immunoprecipitated materials using hFasRECD-T-Fc (N102Q, N120Q) mutant; M, Molecular-weight markers. “+CHO” and “ΔCHO” indicate the migration positions of N-glycan untrimmed and N-glycan trimmed tag-free hFasLECD, respectively.

Figure 1 summarizes the system used for the secretory production of recombinant hFasLECDs in this study. As shown in Figure 1a, the gene organization of the expression unit was essentially the same as in the previous study [10]. Figure 1b illustrates the cultivation system using a plastic culture-bag schematically. The necessary air for the growth of *P. pastoris* transformant cells was supplied forcibly with a diaphragm-type vacuum pump. The culture-bag on a stainless-steel holder was rotated in a thermostatic air incubator gently (80-85 rpm) during the cultivation period in order to avoid sedimentation of the *P. pastoris* cells. The dimensions of the culture bag and the air ventilation speed were 190 mm × 190 mm × 190 mm and 2.5 liter/min, respectively. In Table 1, the purification course of tag-free hFasLECD (Figure 1a) produced using the culture-bag system and its N-glycan trimmed derivative is presented. The purification of tag-free hFasLECD was performed through two rounds of cation-exchange chromatography (Figure 2a). Starting from 2300 ml of the recovered supernatant after 96 h cultivation, the final 29.4 mg yield of highly purified tag-free hFasLECD sample (Figure 2c, solid line and Figure 2d, lane a) was obtained. This purification yield (12.8 mg per liter) corresponded to a three-fold increase as compared with the previously reported purification yield (4.2 mg per liter) concerning a sample of the same quality, which was obtained from a total of 4000 ml culture supernatant produced by 8 rounds of the 500 ml scale cultivation using a 3000 ml volume of glass baffled culture-flask as reported previously [14].

**Table 1:** Purification course of tag-free hFasLECD and its N-glycan trimmed derivative

**Purification step**	**Sample volume (ml)**	**Total protein (mg)**
Culture supernatant	2300	N. D.^*1^
1^st^ Ultrafiltration plus buffer exchange	166	3320
1^st^ Cation-exchange chromatography and 2^nd^ ultrafiltration plus buffer exchange	10.5	54.2
2^nd^ Cation-exchange chromatography	196^*2^	29.4^*2^
Endo Hf treatment, Con A-column fractionation and 3^rd^ Cation-exchange chromatography	43^*3^	10.5^*3^

^*1^Not determined. ^*2^Final N-glycan untrimmed product. ^*3^Final N-glycan trimmed product.

As demonstrated in the previous study concerning FLAG®-(Gly)_5_ tagged sample [10], the remaining N-glycans in tag-free hFasLECD sample in this study could also be trimmed with an Endo H-type glycosidase, Endo Hf (Figure 2d, lane b). In Figure 2b and Figure 2c, the 3rd cation-exchange chromatography profile of the sample after the digestion with Endo Hf and the size-exclusion chromatography profile of the cation-exchange chromatography fractionated sample are shown, respectively. The sample-peak elution-time in the size-exclusion chromatography was substantially delayed after Endo Hf digestion (Figure 2c), which showed the effect of N-glycan trimming on the molecular weight of tag-free hFasLECD. Figure 2d summarizes the purification course during the N-glycan trimming using sodium dodecyl sulfate polyacrylamide gel electrophoresis (SDS-PAGE) analysis (lanes a-d).

**Figure 3:**
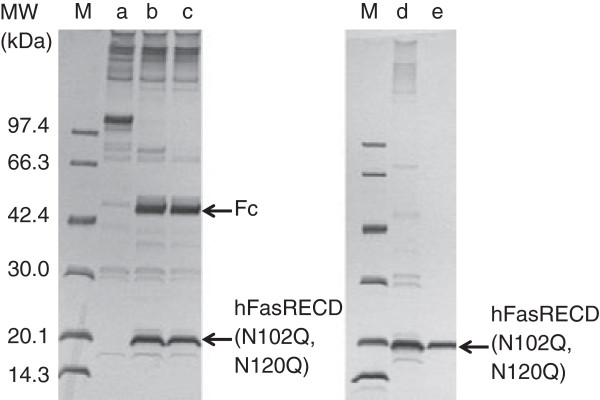
**SDS-PAGE analysis of digestion of hFasRECD-T-Fc (N102Q, N120Q) mutant with thrombin and purification of hFasRECD part.** Protein G-agarose column purified hFasRECD-T-Fc (N102Q, N120Q) mutant (470 μg) was digested with bovine thrombin (47 U) in 50 mM Tris-HCl (pH 7.5) at 20°C, and the liberated mutant hFasRECD part in the reaction mixture was further purified as described previously [15]. Lanes: M, molecular-weight size markers; a, before digestion; b and c, after digestion (b, 2 h; c, 4 h); d, after removal of Fc fragment by Protein G-agarose column; e, after fractionation by cation-exchange chromatography. Used column: Resource S 1 ml.

**Figure 4:**
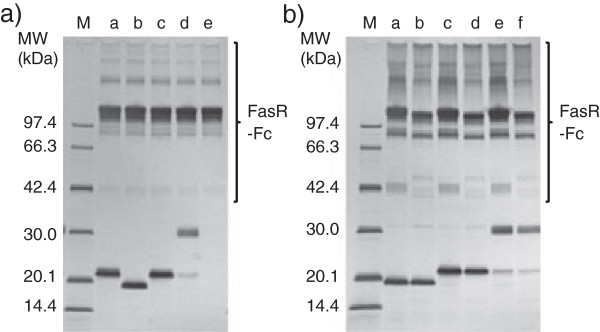
**SDS-PAGE analysis of hFasRECD-T-Fc mediated co-immunoprecipitation of the purified hFasLECDs. a)** Co-immunoprecipitation using wild-type hFasRECD-T-Fc [15]. Lanes: M, molecular-weight size-markers; a, NFG5-hFasLECD [10]; b, tag-free hFasLECD; c, *N*-Ethylmaleimide adduct of NFG1CG4-hFasLECD, d, SUNBRIGHT® ME-050MA adduct of NFG1CG4-hFasLECD; e, buffer alone. The bands in “FasR-Fc” labeled region were derived from wild-type hFasRECD-T-Fc sample. **b)** Comparison between wild-type hFasRECD-T-Fc (lanes a, c and e) and hFasRECD-T-Fc (N102Q, N120Q) mutant (lanes b, d and f). Lanes: M, molecular-weight size-markers; a and b, tag-free hFasLECD; c and d, *N*-Ethylmaleimide adduct of NFG1CG4-hFasLECD; e and f, SUNBRIGHT® ME-050MA adduct of NFG1CG4-hFasLECD. The bands in “FasR-Fc” labeled region were derived from hFasRECD-T-Fc samples.

Receptor-mediated co-immunoprecipitation using wild-type hFasRECD-T-Fc and Protein A-agarose beads was used for the evaluation of the binding activity of the purified hFasLECD samples toward hFasRECD. In this study, the binding activity of the samples was also examined using a mutant hFasRECD-T-Fc, which contains double amino acid residue substitutions (N102Q and N120Q) to remove all possible N-glycosylation sites in the hFasRECD part (Figure 3). In contrast to the presence of several discrete bands in the final purified sample of isolated wild-type hFasRECD part [15], this mutant gave a single band after cleavage with bovine thrombin due to the lack of heterologous N-glycosylation (Figure 3, lane e). Both N-glycan untrimmed and trimmed tag-free hFasLECD samples presented clear binding activity against either wild-type hFasRECD-T-Fc or the N-glycan deficient mutant (Figure 4a, lane b; Figure 4b, lanes a and b; Figure 2d, lanes e and f).

### Secretory expression of NFG1CG4-tagged hFasLECD

**Figure 5:**
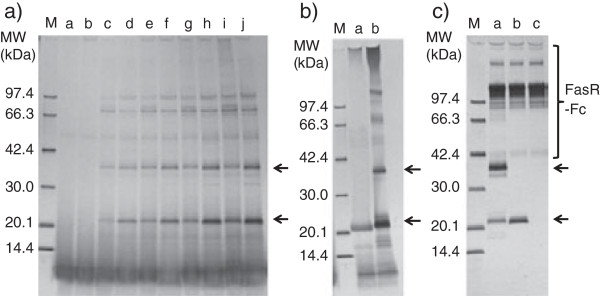
**SDS-PAGE analysis of secretory expression of NFG1CG4-hFasLECD. a)** Comparison of the expression level using glass baffled-flask with that using plastic culture-bag. Lanes: M, molecular-weight size-markers; a and b, at 0 h; c and d, at 24 h; e and f, at 48 h; g and h, at 72 h; i and j, at 96 h. Five μl each samples of culture supernatants either from 500 ml scale culture in 3000 ml baffled flask (lanes a, c, e, g and i) or 2500 ml scale culture in 5000 ml disposable plastic bag (lanes b, d, f, h and j) were applied to each lane. Upper and lower arrows indicate the migration positions of dimeric and monomeric subunits of NFG1CG4-hFasLECD, respectively. **b)** SDS-PAGE analysis of disulfide bond reduction in partially purified NFG1CG4-hFasLECD. Main peak fraction of the *P. pastoris* culture supernatant (29.5°C, 96 h) in 1st cation-exchange column chromatography was concentrated and treated with SDS-PAGE sample buffers with / without 2-mercaptoethanol. Lanes: M, molecular-weight size markers; a, with 2-mercaptoethanol; b, without 2-mercaptoethanol. Upper and lower arrows indicate the same as in **a)**. **c)** Co-immunoprecipitation of the secreted product with wild-type hFasRECD-T-Fc. Lanes: M, molecular-weight size-markers; a, 5 μl culture supernatant of 40-fold concentrated NFG1CG4-hFasLECD from 500 ml scale culture in 3000 ml baffled flask at 96 h; b, purified NFG5-hFasLECD [10] (5 μg); c, buffer alone. Upper and lower arrows indicate the same as in **a)**. The bands in “FasR-Fc” labeled region were derived from wild-type hFasRECD-T-Fc sample.

Based on promising results from the production of tag-free hFasLECD, the secretion of NFG1CG4-hFasLECD (Figure 1a), which should have an additional reactive cysteine residue per subunit of the trimeric molecule within the (Gly)_5_ region in N-terminal FLAG®-(Gly)_5_ tagged hFasLECD (NFG5-hFasLECD) (Figure 1a), was examined for the applicability of the system using a disposable culture-bag. In Figure 5a, a comparison of the secretion level of NFG1CG4-hFasLECD between the cultivation system using the baffled glass flask and that using the disposable plastic bag is shown. As seen from the densities of the corresponding bands in SDS-PAGE analysis, the secretion level was significantly improved in the culture-bag system as compared with the baffled culture-flask system at all sampling times of every 24 h interval within the 96 h cultivation (Figure 5a). The subunits composing secreted NFG1CG4-hFasLECD trimers mainly existed as a couple of forms, monomer and dimer, under disulfide-bridge non-reducing conditions (Figure 5b, lane b). Both forms of NFG1CG4-hFasLECD subunits in the culture supernatant were involved in the specific binding toward wild-type hFasRECD-T-Fc protein (Figure 5c, lane a). The possible inter-subunit disulfide bond formed from the cysteine residues in the dimeric subunits of NFG1CG4-hFasLECD was easily cleaved with 2-mercaptoethanol contained in the reducing sample loading buffer, and single major band of the reduced sample appeared at the position of monomeric form in SDS-PAGE analysis (Figure 5b, lane a).

### Chemical modification of NFG1CG4-hFasLECD with single maleimide group containing compounds

**Figure 6:**
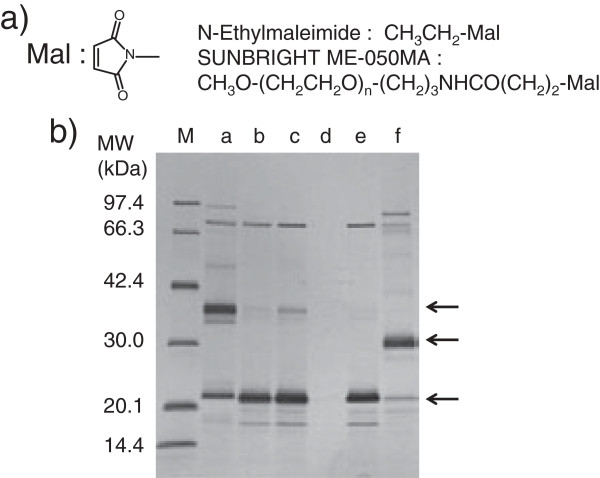
**Reaction of NFG1CG4-hFasLECD with *N*-Ethylmaleimide/SUNBRIGHT® ME-050MA. a)** Chemical structures of *N*-Ethylmaleimide and SUNBRIGHT® ME-050MA. **b)** SDS-PAGE analysis of the reaction mixtures at each step. Lanes: M, molecular-weight size-markers; a, before reaction; b, after reaction with TCEP; c, PD-10 column “Elution fraction”; d, PD-10 column “Wash fraction”; e, after reaction with *N*-Ethylmaleimide followed by quenching with L-Cysteine hydrochloride; f, after reaction with SUNBRIGHT® ME-050MA followed by quenching with L-Cysteine hydrochloride. Upper, middle and lower arrows indicate the migration positions of disulfide-bridged subunits, SUNBRIGHT® ME-050MA adduct and non-conjugated subunit/*N*-Ethylmaleimide adduct of NFG1CG4-hFasLECD, respectively.

The secreted NFG1CG4-hFasLECD was first partially purified using the same cation-exchange chromatography as described for tag-free hFasLECD, and then treated with moderate concentration of Tris-(2-carboxyethyl)phosphine (TCEP) under neutral pH at room temperature. This TCEP reduced product was immediately subjected to the chemical modification reaction with two kinds of single maleimide-group containing compounds, i. e. *N*-Ethylmaleimide and SUNBRIGHT® ME-050MA. SUNBRIGHT® ME-050MA is a mono-functional linear methoxy polyethylene glycol (PEG), which has the molecular-weight of approximately 5 kDa and contains an active maleimide group at one end (Figure 6a). In Figure 6b, SDS-PAGE analysis of the reaction mixtures at each individual step is shown. After the treatment of partially purified NFG1CG4-hFasLECD (lane a) with 20 mM TCEP for 1 h at room temperature, the possible inter-subunit disulfide bond was completely cleaved (lane b). This disulfide bond was slightly regenerated during the desalting step to remove excess amount of TCEP (lane c). However, the original inter-subunit disulfide bond was not observed in the reaction mixture with an excess amount of single maleimide-group containing compounds (lanes e and f).

**Figure 7:**
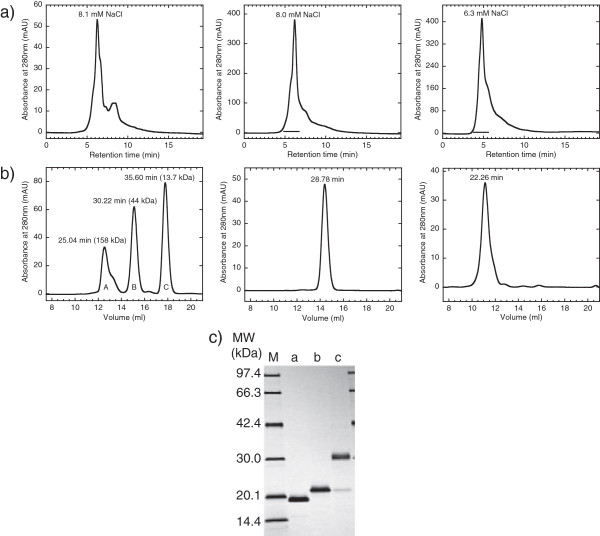
**Purification of NFG1CG4-hFasLECD conjugated with *N*-Ethylmaleimide/SUNBRIGHT® ME-050MA. a)** Elution profiles in cation-exchange chromatography. Used column: Resource S 1 ml. Panels: left, before reaction (non-reduced); center, after reaction with *N*-Ethylmaleimide and quenching; right, after reaction with SUNBRIGHT® ME-050MA and quenching. The fractions shown in underbars were collected as the final products. NaCl concentrations under each principal peak eluting condition are described. **b)** Elution profiles of fractionated products in size-exclusion chromatography. Used column: Superdex 200 10/300 GL. Elution buffer: 50 mM sodium acetate plus 150 mM NaCl (pH 5.6). Flow rate: 0.5 ml/min. Panels: left, molecular-weight standards mixture (A, Aldolase; B, Ovalbumin; C, Ribonuclease A); center, *N*-Ethylmaleimide modified product; right, SUNBRIGHT® ME-050MA modified product. Retention times of each peak are described. **c)** SDS-PAGE analysis of fractionated products. Lanes: M, molecular-weight size-markers; a, tag-free hFasLECD (0.15 μg); b, *N*-Ethylmaleimide adduct of NFG1CG4-hFasLECD (0.15 μg); c, SUNBRIGHT® ME-050MA adduct of NFG1CG4-hFasLECD (0.15 μg).

The purification of the NFG1CG4-hFasLECD modified with single maleimide-group containing compounds was performed by cation-exchange column chromatography (Figure 7a, center and right panels). The less sharp peak following the main peak observed in the sample before the conjugation reaction (Figure 7a, left panel) virtually disappeared from the sample after modifications. The sodium chloride (NaCl) concentrations required for elution of the chemically modified products depended on the molecular structure of the maleimide reagent. The main peak of *N*-Ethylmaleimide conjugated NFG1CG4-hFasLECD was eluted under almost the same NaCl concentration (8.0 mM) as that for the main peak of the sample before the conjugation reaction (8.1 mM). The main peak of SUNBRIGHT® ME-050MA conjugated NFG1CG4-hFasLECD was eluted under significantly lower NaCl concentration (6.3 mM), suggesting weaker binding to a negatively charged column carrier due to the reduced ionic interactions caused by the possible shielding effect of the attached PEG moieties.

In order to probe the assembly state and the molecular shape of each modified product, the fractionated samples in the above cation-exchange chromatography were subjected to analysis using a size-exclusion chromatography (Figure 7b). Either sample presented a symmetrical single peak, suggesting the uniformity of molecular-weight of the fractionated products (Figure 7b, center and right panels). The peak retention time of the *N*-Ethylmaleimide conjugated sample (28.78 min) was between the size-markers of 44 kDa (30.22 min) and 158 kDa (25.04 min) (Figure 7b, left panel), and was nearly the same as observed for tag-free hFasLECD (29.55 min) under the identical elution condition using the same chromatography column (Figure 2c, solid line). This indicated that no significant change in the molecular conformation occurred to hFasLECD by the conjugation of *N*-Ethylmaleimide. On the other hand, SUNBRIGHT® ME-050MA conjugated sample eluted markedly early (22.26 min) and the peak retention time was even significantly earlier than the 158 kDa size-marker (25.04 min). This suggested that the PEG moieties attached to the N-terminal tag sequence region of NFG1CG4-hFasLECD had a much more extended conformation to behave as a molecule with virtually bigger molecular weight, which rendered the retention time markedly earlier than the expected value from its actual calculated molecular-weight (ca. 70 kDa as the triply modified product).

Figure 7c shows the SDS-PAGE analysis of the fractionated samples. As shown in lane b, the *N*-Ethylmaleimide conjugated NFG1CG4-hFasLECD sample exhibited a single band at the position of approximately 21 kDa. On the other hand, the SUNBRIGHT® ME-050MA conjugated NFG1CG4-hFasLECD sample exhibited two bands, which consisted of one major thick band migrated at approximately 30 kDa and another minor faint band at 21 kDa (Figure 7c, lane c). This result suggested that greatest portions of free cysteine residues in the NFG1CG4-hFasLECD sample were conjugated with SUNBRIGHT® ME-050MA moieties. Table 2 summarizes the purification course of NFG1CG4-hFasLECD conjugated with the single maleimide group containing compounds. Starting from 2500 ml of the culture supernatant, 24.5 mg and 16.5 mg of the purified products were recovered with respect to the *N*-Ethylmaleimide adduct and the SUNBRIGHT® ME-050MA adduct, respectively.

**Table 2:** Purification course of NFG1CG4-hFasLECD conjugated with single maleimide group containing compounds

**Purification step**	**Sample volume (ml)**		**Total protein (mg)**	
Culture supernatant	2500		N. D.^*1^	
1^st^ Ultrafiltration plus buffer-exchange	140		3024	
1^st^ Cation-exchange chromatography and 2^nd^ ultrafiltration plus buffer-exchange	7.0		69.4	
After chemical modification reaction and 2^nd^ cation-exchange chromatography^*2^	100^*3^	108^*4^	24.5^*3^	16.5^*4^

^*1^Not determined. ^*2^Normalized to the values starting from initial sample volume (2500 ml). ^*3^Final *N*-Ethylmaleimide conjugated product. ^*4^Final SUNBRIGHT® ME-050-MA conjugated product.

The purified samples conjugated with single maleimide group containing compounds exhibited comparable binding activity to that of NFG5-hFasLECD toward wild type hFasRECD-T-Fc (Figure 4a, lanes a, c and d). Both bands in the SUNBRIGHT® ME-050MA conjugated sample were found in the materials co-immunoprecipitated with wild-type hFasRECD-T-Fc protein. Therefore, the minor band was considered to be derived from a small fraction of the functional trimeric product containing the subunit component with the remaining non-conjugated cysteine residue. The comparison of the data using wild-type hFasRECD-T-Fc with that of the N-glycan deficient hFasRECD-T-Fc mutant revealed that the binding activity toward this mutant was essentially maintained with all examined hFasLECD samples (Figure 4b), however the SUNBRIGHT® ME-050MA conjugated sample had a reduced binding strength toward the mutant hFasRECD-T-Fc than the wild-type protein judging from the evident but weaker density of the bands of the co-immunoprecipitated materials (Figure 4b, lanes e and f).

### Cross-linking of NFG1CG4-hFasLECD with two maleimide-groups containing polyethylene glycol

**Figure 8:**
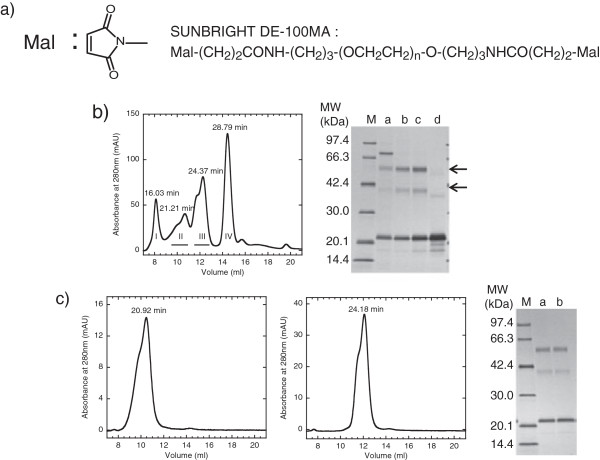
**Chemical modification of NFG1CG4-hFasLECD with SUNBRIGHT® DE-100MA. a)** Chemical structure of SUNBRIGHT® DE-100MA. **b)** Fractionation of the reaction mixture using size-exclusion chromatography. Used column: Superdex 200 10/300 GL. Elution buffer: 50 mM sodium acetate plus 150 mM NaCl (pH 5.7). Flow rate: 0.5 ml/min. Panels: left, elution profile; right, SDS-PAGE analysis of each peak fraction. Retention times of each peak are described (left panel). The fractions shown in underbars were collected as the final products. Lanes: a, peak I; b, peak II; c, peak III; d, peak IV (right panel). The protein bands composed only of the NFG1CG4-hFasLECD subunits conjugated with SUNBRIGHT® DE-100MA are arrowed. **c)** Characterization of the fractionated products. Panels: left and center, elution profiles of peak II (left) and peak III (center). Retention times of each peak are described. Right panel, SDS-PAGE analysis. Lanes: M, molecular-weight size-markers; a, peak II fraction (0.3 μg); b, peak III fraction (0.3 μg).

Partially purified NFG1CG4-hFasLECD reduced with moderated concentration of TCEP under neutral pH at room temperature was also examined for cross-linking with two maleimide groups containing compound, SUNBRIGHT® DE-100MA. SUNBRIGHT® DE-100MA is a homo-bifunctional linear PEG with the molecular-weight of approximately 10 kDa, which has two maleimide groups at either end of the molecule (Figure 8a). In Figure 8b, an elution profile of the reaction mixture after the cross-linking reaction on a size-exclusion chromatography column and SDS-PAGE analysis of the fractionated peaks in the size-exclusion chromatography are displayed. Peaks, I, II and III contained the cross-linked materials. The band with the highest molecular-weight between molecular size-markers of 66.3 kDa and 97.4 kDa found in the peak I sample (Figure 8b, right panel, lane a) was regarded to correspond to the major impurity protein found in the sample before the cross-linking reaction (Figure 6b, lane c). From the molecular-weight of the bands, the upper arrowed band and lower arrowed band found in peaks I, II and III (Figure 8b, right panel, lanes a, b and c) were considered to be two NFG1CG4-hFasLECD subunits cross-linked by one SUNBRIGHT® DE-100MA molecule and one NFG1CG4-hFasLECD subunit conjugated with one SUNBRIGHT® DE-100MA molecule, respectively. The purification yields of the chemically modified products contained in peaks II and III were 7.6 mg and 12.0 mg, respectively, which were obtained from 2500 ml of the culture supernatant of NFG1CG4-hFasLECD.

**Figure 9:**
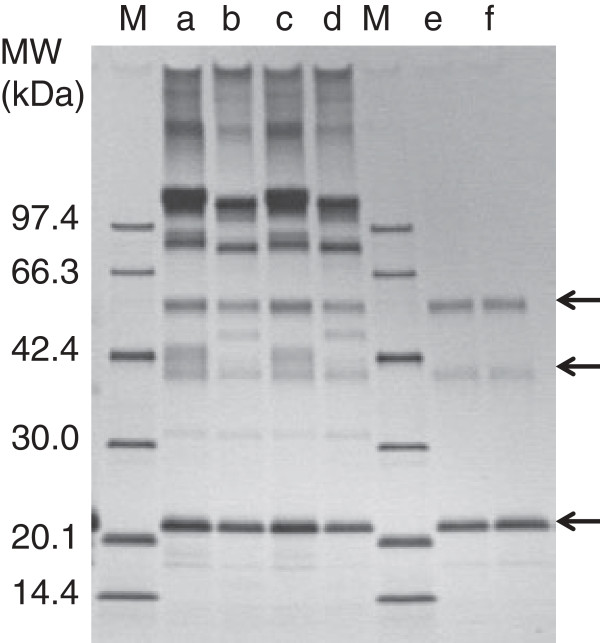
**SDS-PAGE analysis of hFasRECD-T-Fc mediated co-immunoprecipitation of the fractionated hFasLECDs cross-linked with SUNBRIGHT® DE-100MA.** Either wild-type hFasRECD-T-Fc (lanes a and c) or hFasRECD-T-Fc (N102Q, N120Q) mutant (lanes b and d) was used for the experiment. Lanes: M, molecular-weight size markers; a and b, peak II (co-immunoprecipitated sample); c and d, peak III (co-immunoprecipitated sample); e, peak II (fractionated sample, 0.3 μg); f, peak III (fractionated sample, 0.3 μg). The components in the peaks II and III are arrowed.

The samples fractionated as peaks II and III were independently re-chromatographed on the same column again (Figure 8c, left and center panels). Both peaks held approximately the same original retention time, suggesting that the product in peak II was not just a transient aggregation of the product in peak III. The same amounts of purified proteins were subjected to SDS-PAGE analysis (Figure 8c, right panel). This revealed that peak II and peak III contained essentially the same subunit components with identical compositions. Materials in peak II were considered as an additional associated product of materials in peak III, which were composed of more highly cross-linked NFG1CG4-hFasLECD. All components in peaks II and III were found in the precipitated samples obtained in the receptor-mediated co-immunoprecipitaion experiments using either wild-type or the mutant hFasRECD-T-Fc (Figure 9), which suggested that the cross-linked products in peaks II and III retained the binding activity toward hFasRECD.

### Cytotoxic activity of chemically modified NFG1CG4-hFasLECD

**Figure 10:**
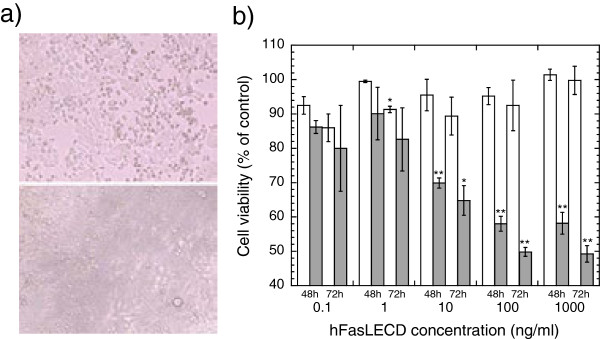
**Cytotoxic activity of *N*-Ethylmaleimide conjugated NFG1CG4-hFasLECD against HT-29 cells. a)** Effect on cell morphology. Panels: upper, 72 h treatment with *N*-Ethylmaleimide conjugated NFG1CG4-hFasLECD sample (100 ng/ml); lower, 72 h treatment with buffer alone sample. Either treatment was conducted in the presence of anti-FLAG® M2 antibody (2 μg/ml). **b)** Effect of sample concentration and treatment time. Symbols: white bars, in the absence of the M2 antibody; gray bars, in the presence of the M2 antibody. Standard error of mean under each experimental condition was included as an error bar. *(p < 0.05) and **(p < 0.01) show comparisons with the control experiments using Student’s t-test.

In order to examine the cytotoxic activity of NFG1CG4-hFasLECD containing site-specific modification with a maleimide compound, the cell viability of a human colorectal adenocarcinoma HT-29 cell line was evaluated using 3-(4, 5 Dimethylthiazol-2-yl 2, 5-diphenyl-tetrazolium bromide (MTT) assay. The susceptibility enhancement in apoptosis induction by pretreatment with interferon-γ concerning this cell line [16] was not applied here. Figure 10a shows the morphologic change of HT-29 cells induced by treatment with *N*-Ethylmaleimide conjugated NFG1CG4-hFasLECD, indicating the occurrence of apoptosis. A number of apparently dark and shrank cells suggesting the occurrence of apoptosis, which were not present in the sample at 72 h treatment by buffer alone (lower), were observed in the micrograph of the sample at 72 h treatment by 100 ng/ml of *N*-Ethylmaleimide conjugated NFG1CG4-hFasLECD (upper). In Figure 10b, the cytotoxic effect of the purified *N*-Ethylmaleimide conjugated NFG1CG4-hFasLECD on HT-29 cells in the presence or absence of anti-FLAG® M2 antibody is shown. The Fas ligand sample exhibited significant cell-death inducing activity in a dose and time dependent manner in the presence of the M2 antibody. The buffer alone sample in vehicle experiment showed no significant activity even after 72 h treatment under either condition, and the cell viability (mean ± standard deviation) in the presence and absence of the M2 antibody was 95.3 ± 1.4% and 103 ± 3.4% of control, respectively. The above results suggested the induction of apoptosis via Fas ligation by *N*-Ethylmaleimide conjugated NFG1CG4-hFasLECD was evident only after cross-linking through the recognition of FLAG® sequence existing in the NFG1CG4-tag by M2 antibody.

## Discussion

Heterologous production systems of recombinant human Fas ligand extracellular domain have been developed using several expression hosts. It has been shown that *P. pastoris* and *D. discoideum* were the most efficient hosts in providing functional products by direct secretion without the necessity of fusion with other protein domains [6]. A methanol utilization plus strain, *P. pastoris* GS115 (Mut^+^), has been used as the host in the secretory production of this protein [8-10,14]. The best purification yield of 24.3 mg/l was reported with the NFG5-hFasLECD (aa 139-281) containing the mutations of N184Q and N250Q [10]. However, the deletion of NFG5 tag sequence in this mutant resulted in a large reduction of the purification yield to 4.2 mg/l [14]. In this study, it was shown that the purification yield of tag-free hFasLECD could be increased 3-fold by altering the cultivation vessel used in the production system from glass baffled flask to disposable plastic bag. Good aeration during cultivation in expression induction-phase using methanol is a critical factor in the production efficiency of the recombinant proteins in *P. pastoris*. The cell density of another species of yeast, *Saccharomyces cerevisiae,* was significantly increased in cultivation using the plastic culture-bag compared with that using baffled flask (Y. Kawabata, personal communications). In another experiment for the production of NFG1CG4-hFasLECD in *P. pastoris* GS115 strain using 4000 ml BMMY medium in a 10 liter volume plastic culture-bag (CB-10; Fujimori Kogyo), an approximately 50% increase in optical density at 600 nm was obtained compared with the corresponding cultivation using 50 ml BMMY medium in 500 ml triple side-baffled glass flask after 96 h cultivation (unpublished result). Therefore, the main reason for the yield enhancement in this study may be ascribed to better aeration by the forced air ventilation using a diaphragm pump and increase of cell density in the cultivation.

Single-use technologies using disposable apparatus including plastic bags are currently evolving industrial technologies, which have a number of merits in the production of recombinant proteins, especially in the field of bio-pharmaceutical manufacturing [17]. One of the important areas of the application of single-use technologies is fermentation of microorganisms including *P. pastoris*. In this study, it was demonstrated that the use of a disposable plastic bag was also effective for the purpose of enhancing the yield of recombinant proteins aiming at the research of its site-specific modification using *P. pastoris* as the producer. To date, remarkable increases in the yield of recombinant proteins from shake-flask systems have been achieved in many cases by non-disposable system such as a jar fermenter, which are mostly made of glass and stainless-steel [18]. This study demonstrated that the alternative possibility of enhancing the yield using disposable plastic culture-bag system, though the increased level was moderate as compared with a reusable fermenter system operated under optimized conditions. Alteration of the cultivation vessel provided not only an increase in the purification yield per unit volume of the culture supernatant but also reduction of the number of vessels necessary for the same total cultivation volume. Moreover, the plastic culture-bag system requires neither any special ingredient in the medium nor expensive equipment for cultivation, making this system convenient compared with a fermenter system, which requires special materials for cultivation. The culture-bag system should be readily applicable to production of other recombinant proteins in yeasts on a small laboratory scale.

The interactions between hFasLECD and hFasRECD constitute a key signaling step in the initiation of apoptotic processes via extrinsic pathway mediated by human Fas receptor. Some aspects of the interactions have been characterized through the site-directed mutagenesis studies [19,20] and the three-dimensional structures produced by *in silico* modeling [21,22]. Either tag-free hFasLECD containing untrimmed or trimmed N-glycan exhibited virtually identical binding activity toward mutant hFasRECD-T-Fc lacking N-glycosylation site within the hFasRECD part with the activity toward wild type hFasRECD-T-Fc, suggesting N-glycosylation in both hFasLECD and hFasRECD is not a prerequisite for protein – protein interactions between hFasLECD and hFasRECD.

Site-specific chemical modifications provide a useful method to obtain proteins with improved pharmaceutical properties by enhancing *in vivo* biological efficacy. A number of therapeutic proteins with site-specific modifications, especially pegylation, have been developed [23]. Suitable selection of the structural position for modification is essential for effective derivation, while maintaining the intrinsic biological functions of the target proteins. The N-terminal region of NFG1CG4-hFasLECD is thought to be located distant from the binding interface to hFasRECD judging from a detailed three-dimensional structure of the complex between hFasLECD and a decoy receptor, DcR3 [24].

NFG5-hFasLECD (aa 139-281, N184Q, N250Q) is considered to contain a single disulfide bond between Cys 202 and Cys 233 within a structurally buried region by analogy with the available three-dimensional structure of hFasLECD [24]. However, it is known that TCEP can selectively reduce solvent exposed disulfide bonds under mild reaction conditions without affecting the structurally buried one [25]. Therefore, the N-terminal tag sequence region in NFG5-hFasLECD [9] was chosen as the introduction site of solvent accessible cysteine residue for site-specific chemical modifications in this study. A free cysteine residue is ideal for selective modifications due to its unique reactive properties against many electorophiles [25,26]. The maleimide group containing compound is useful, since a variety of commercial compounds including those for pegylation are readily available with reasonable prices. It was found that NFG1CG4-tag sequence, FLAG®-GlyCys(Gly)_4_, effectively worked as a new N-terminal tag for site-specific chemical modifications, which provided an alternative to existing methods for N-terminal conjugation using different tag sequences [27,28].

The *N*-Ethylmaleimide conjugated NFG1CG4-hFasLECD exhibited a cell-death inducing activity against HT-29 cells, a human colorectal adenocarcinoma cell line. The cytotoxic activity was significant only after cross-linking by M2 antibody specific for FLAG®-tag as depicted with the activity of a FLAG®-tagged trimeric hFasLECD against Jurkat, Raji and HeLa cells [29]. This suggested that site-specific chemical modifications via conjugation of the cysteine residue in the *N*-terminal NFG1CG4-tag with maleimide compounds were possible without losing the intrinsic pro-apoptotic function of the sample produced in *P. pastoris*[8]. This result constitutes the basis of development of novel type cytotoxic therapeutic agents using site-specific chemical modifications with targeting moieties such as tumor antigen specific single chain antibody [30,31]. This also showed that the two N-glycan chains at Asn184 and Asn250 sites were not always essential for exhibiting the cell-death inducing activity.

To our knowledge, this is the first report describing preparations of functional hFasLECD samples with site-specific chemical modifications. Human FasLECD works as a pro-apoptotic receptor agonist by binding to hFasRECD on the plasma membrane of target cells. However, it also binds to DcR3 with a similar affinity to hFasRECD at the same time [32], which can be one of the possible reasons leading to onset of diseases caused by excessive cellular proliferation [33]. Overexpression of DcR3 decoy receptor was found in several types of cancers and autoimmune diseases [34]. Protein engineering studies including the site-specific chemical modifications might contribute to solve this problem by tuning the receptor binding specificity of hFasLECD.

It has been shown that a hexameric, genetically fused proteins containing two trimers of hFasLECD within the assembled molecule via the association of ACRP30 collagen domain region, displayed much higher killing activity than trimeric hFasLECD against several kinds of tumor cells [29]. The site-specific cross-linking strategy concerning hFasLECD demonstrated in this study might also contribute to the development of such engineered molecules with enhanced cell-killing activity.

Possible applications aimed at membrane bound forms of human Fas receptor on the targeted cells include *in vivo* / *in vitro* imaging of positive cells [35] by conjugations with either fluorescent dyes [36] or luminescent proteins [37]. It is also known that soluble agonistic and decoy receptor proteins concerning human Fas receptor system are useful biomarkers in serum, urine and other body fluids for early diagnosis [38], prognosis [39,40], response to drug treatment [41] and mortality [42] of many serious human diseases represented by cancers. The mutant hFasLECD containing the reactive cysteine residue conjugable to maleimide group containing compounds should also become a powerful molecular agent in developing devices for quantifying such disease specific biomarkers.

## Conclusions

In the present study, a new, convenient and efficient production system by *P. pastoris* using a disposable plastic culture-bag was developed, which requires neither special ingredients in the culture medium nor expensive equipment such as a jar fermenter. Using this system, the purification yield of tag-free hFasLECD increased three-fold. This system was also applicable to the secretory production of a mutant hFasLECD, which was appropriate for the site-specific conjugations with maleimide group containing compounds. The enhanced yield will facilitate further chemical characterization studies on hFasLECD. The conjugated hFasLECDs with maleimide group containing compounds at its N-terminal tag sequence showed receptor binding and cell-death inducing activities. The site-specific chemical modifications of hFasLECD should contribute to development of novel therapeutic agents as well as tools for diagnostic purposes in the biomedical field.

## Methods

### Materials

Plasmid vectors, pNFG5-hFasLECD (aa 139-281) with (N184Q, N250Q) double mutations and its tag-free version were prepared as described in previous papers [10,14]. The insertion mutation for the introduction of the additional cysteine residue in NFG1CG4-hFasLECD (Figure 1a) was conducted by *in vitro* mutagenesis of NFG5-hFasLECD gene as a custom service by Takara-bio Co. *Pichia pastoris* GS115 (Mut^+^) was used as the strain for expression. Buffered glycerol complex medium (BMGY medium) and buffered methanol complex medium (BMMY medium) were prepared as described [9]. Triple side-baffled culture flasks were purchased from Asahi Glass Co., Ltd. Disposable plastic culture-bags (CB-5) and a stainless-steel bag-holder (Bag-holder 10) were products of Fujimori Kogyo Co., Ltd. NFG5-hFasLECD protein was obtained as described previously [10]. Wild-type and (N102Q, N120Q) mutant hFasRECD-T-Fc proteins were obtained using baculovirus-silkworm expression system [15]. The 10-20% gradient gels used for SDS-PAGE analysis and L-Cysteine hydrochloride monohydrate were purchased from Wako Pure Chemical Ind., Ltd. Culture filtration-devices and tangential flow filtration-devices for concentration were obtained from Nihon Pall, Ltd. Cation-exchange chromatography and size-exclusion chromatography were performed using columns and devices from GE healthcare. BCA protein assay kit and TCEP neutral pH solution were purchased from Thermo Fisher Scientific Inc. SUNBRIGHT® ME-050MA: α-[3-(3-Maleimido-1-oxopropyl) amino]propyl-ω-methoxy, polyoxyethylene (5 kDa fraction, 98.1%; average molecular weight, 5393; polydispersity, 1.02; maleimide group content, 95.0%) and SUNBRIGHT® DE-100MA: α-[3-(3-Maleimido-1-oxopropyl)amino]propyl-ω-[3-(3-Maleimido-1-oxopropyl)amino]propoxy, polyoxyethylene (10 kDa fraction, 96.0%; average molecular weight, 10644; polydispersity, 1.02; terminal activated rate, 83.9%) were obtained from NOF Co. *N*-Ethylmaleimide and Phosphate buffer solution (pH 6.4) were from Nakarai Tesque. Immunoprecipitation kit (Protein A) was from Roche Diagnostics. Other chemical reagents of analytical grade and devices used for protein purification were as described [9]. Chemical structure of maleimide group was drawn using Accelrys Draw 4.1.

### Production of tag-free hFasLECD using baffled glass culture-flask

The experimental procedures used during the selection of efficient single colonies of the recombinant *P. pastoris* and those used for the secretory production of tag-free hFasLECD in 500 ml scale culture of BMMY medium (pH 6.2-6.5) using a 3000 ml baffled flask made of borosilicate glass were the same as described previously [15]. Cultivation was conducted at 29.5°C with a rotation of 300 rpm in a thermostatic air incubator (BR-32FL; TAITEC).

### Expression of tag-free and NFG1CG4-hFasLECDs using disposable plastic culture-bag

The same BMMY medium for the above baffled culture-flask was used in the cultivation of *P. pastoris* transformant using a disposable culture-bag made of polypropylene. The *P. pastoris* pre-culture was prepared by cultivation in 500 ml BMGY medium (pH 6.2-6.5) at 29.5°C, 300 rpm overnight using 3000 ml triple side-baffled glass flask. The pre-culture was centrifuged at 8000 rpm for 2 min at room temperature to give the seed cell-pellets for the inoculation of 2500 ml BMMY medium made of either autoclaved or filter-sterilized components in 5000 ml volume of the disposable culture bag. The cultivation was conducted at 29.5°C with a rotation of 80-85 rpm for 96 h in the same thermostatic air incubator as described above. The induction of expression was made by the addition of 0.5% methanol at 24 h intervals.

### Purification of secreted products

The culture medium containing the secreted hFasLECDs was centrifuged and the supernatant was sterilely filtered with a double (0.8 μm and 0.2 μm) poly-ethersulfone membrane. The filtrate was transferred to an ultrafiltration device equipped with a poly-ethersulfone membrane for tangential flow filtration (Molecular-weight cut off: 10 kDa) to concentrate to approximately 100 ml. The concentrated retentate was further buffer-exchanged using 50 mM sodium acetate (pH 5.6). The buffer-exchanged solution was then loaded on a Hi-Trap S cation-exchange column (5 ml) equilibrated with 50 mM sodium acetate buffer (pH 5.6). The recombinant protein was eluted with either 500 mM NaCl (for tag-free hFasLECD) or 300 mM NaCl (for NFG1CG4-hFasLECD). The eluted samples were concentrated using an Amicon Ultra-15 ultrafiltration device (10 kDa) to ca. 4 ml, and further desalted with PD-10 column (8.3 ml) using 50 mM sodium acetate buffer (pH 5.6).

With respect to tag-free hFasLECD, the desalted solution was loaded on a Resource S cation-exchange column (6 ml) equilibrated with 50 mM sodium acetate buffer (pH 5.6). The recombinant proteins were eluted with a linear salt gradient from 50 to 450 mM NaCl in 50 mM sodium acetate buffer (pH 5.2) at the flow rate of 6 ml/min. The fractions containing the recombinant hFasLECD were pooled as the final product. The protein concentration of the samples at each purification step was determined by a BCA protein assay kit using bovine serum albumin as a standard. As for NFG1CG4-hFasLECD, the protein concentration of the desalted solution after the purification step with the Hi-Trap S 5 ml column was determined to be 9.9 mg/ml, and was directly used for the reaction with maleimide group containing compounds.

### Preparation of N-glycan trimmed tag-free hFasLECD

Purified sample of tag-free hFasLECD was concentrated to 1.92 mg/ml with Amicon Ultra 15 (Molecular weight cut-off: 10 kDa), and digested with Endo Hf (New England Biolabs, Inc.). Twelve μl of 500 mM sodium citrate buffer (pH 5.5) was added to 192 μg of tag-free hFasLECD sample and then treated with 12000 U of Endo Hf at 37°C, for 48 h. The N-glycan trimmed tag-free hFasLECD in the reaction mixture was purified according to essentially the same procedures as described for NFG5-hFasLECD [10]. In brief, the above Endo Hf reaction mixture was first subjected to Con A-agarose column to trap the N-glycan untrimmed tag-free hFasLECD, and the flow-through fraction containing the N-glycan trimmed tag-free hFasLECD was then further fractionated by Mono S 1 ml column cation-exchange chromatography using the elution with a linear salt gradient from 50 to 550 mM NaCl in 50 mM sodium acetate buffer (pH 5.6).

### Reaction of NFG1CG4-hFasLECD with maleimide group containing compounds

The reactions of NFG1CG4-hFasLECD sample with single maleimide group containing compounds (*N*-Ethylmaleimide and SUNBRIGHT® ME-050MA) were conducted as follows. An aliquot of the sample solution (1.6 ml) containing 15.8 mg protein was mixed with 16 μl of 0.5 M Ethylenediaminetetraacetic acid sodium salt (EDTA Na) solution (pH 8.0) and then treated with 64 μl of 0.5 M TCEP solution (neutral pH) for 1 h at 27°C. The reaction mixture was resolved by a PD-10 desalting column (8.3 ml) to remove excess amount of TCEP using 25 mM phosphate buffer plus 2 mM EDTA Na (pH 6.4) as the eluent. After the loading of the sample in a total volume of 2.5 ml into the column, 3.5 ml of the buffer followed by another 2.5 ml of the buffer was added to collect the flow-through. The former 3.5 ml and the latter 2.5 ml flow-through fractions were named “Elution fraction” and “Wash fraction”, respectively. Virtually, no reduced product was found in the “Wash fraction” (Figure 6b, lane d). The “Elution fraction” was divided into two 1.75 ml parts equally. Either freshly prepared 30 μl of 1 M *N*-Ethylmaleimide solution in ethanol or 189 mg of solid SUNBRIGHT® ME-050MA powder was added to each solution. After 15 min at 27°C, 33 μl of 1 M L-Cysteine hydrochloride solution in water was added to each reaction mixture, and further incubated at 27°C for 15 min in order to quench the excess maleimide groups.

The reaction of NFG1CG4-hFasLECD sample with SUNBRIGHT® DE-100MA was performed as follows. An aliquot of the sample solution (1.6 ml) containing 15.8 mg protein was first treated with TCEP in the same way as described for the reaction with single maleimide group containing compounds. Then, 100 μl of freshly prepared SUNBRIGHT® DE-100MA solution (80 mg in 5 ml) in 25 mM phosphate buffer plus 2 mM EDTA Na (pH 6.4) was added to the PD-10 column “Elution fraction” (3.5 ml), and incubated for 1 h at 22°C. Seventy μl of 1 M *N*-Ethylmaleimide solution in ethanol was added to the reaction mixture and was incubated for 15 min at 22°C to cap the unreacted free cysteine residues in the NFG1CG4-hFasLECD sample. After that, 69 μl of 1 M L-Cysteine hydrochloride was added and incubated for a further 15 min at 22°C to quench the excess maleimide groups.

### Purification of chemically modified NFG1CG4-hFasLECD

The final reaction mixtures concerning the chemical modification of NFG1CG4-hFasLECD with single maleimide group containing compounds were buffer-exchanged with 50 mM sodium acetate solution (pH 5.3) using PD-10 desalting column (8.3 ml). This solution was loaded on a Resource S cation-exchange column (1 ml) equilibrated with the same buffer. The recombinant protein was resolved with a linear salt gradient from 0 to 250 mM NaCl in 50 mM sodium acetate buffer (pH 5.3) at the flow rate of 1 ml/min. The main peak fractions containing the chemically modified NFG1CG4-hFasLECD were pooled, and the product yield was quantified by BCA protein assay.

As for the reaction product of NFG1CG4-hFasLECD with SUNBRIGHT® DE-100MA, the final reaction mixture was first desalted using PD-10 column (8.3 ml) and then concentrated to ca. 1.0 ml with Amicon Ultra 8 ultrafiltration device (Molecular-weight cut off: 10 kDa). An aliquot (230 μl) of the concentrated sample was resolved by a Superdex 200 10/30 GL size-exclusion column (diameter, 10 mm; length, 300 mm) using 50 mM sodium acetate plus 150 mM NaCl (pH 5.6) elution buffer at the flow rate of 0.5 ml/min. The samples of each peak fraction were analysed by SDS-PAGE. The second and the third peak fractions containing inter-molecularly cross-linked NFG1CG4-hFasLECD products were pooled, and the recovery yields were quantified by BCA protein assay.

### Molecular-weight estimation using size-exclusion chromatography

Molecular-weight of the purified samples was estimated by a Superdex 200 10/30 GL size-exclusion column (diameter, 10 mm; length, 300 mm) using the same elution conditions as described above. Fifty five μg each of the untrimmed and N-glycan trimmed tag-free hFasLECD, 35 μg each of the NFG1CG4-hFasLECD modified with single maleimide group containing compounds (*N*-Ethylmaleimide and SUNBRIGHT® ME-050MA), or 230 μl each pooled fractions of the NFG1CG4-hFasLECD cross-linked with SUNBRIGHT® DE-100MA were subjected to chromatography. A mixture of molecular-weight standard proteins (Aldolase, 158 kDa; Ovalbumin, 44 kDa and Ribonuclease A, 13.7 kDa) was used as size-markers.

### Receptor-mediated co-immunoprecipitation of hFasLECD

Detection of the binding activity of hFasLECD samples toward Fas receptor was performed by receptor-mediated ligand immunoprecipitation assay using hFasRECD-T-Fc proteins and Protein A-agarose beads. A commercially available immunoprecipitation kit was used for the assay. The experimental procedures were the same as described in the previous paper [10]. Purified NFG5-hFasLECD protein was employed as the authentic positive control sample.

### Cell culture and cytotoxicity assay

Human colorectal adenocarcinoma cell line HT-29 cells (catalog no. HTB-38) were purchased from ATCC, and maintained in Dulbecco’s modified Eagle’s medium (DMEM; Sigma) supplemented with 10% fetal bovine serum (Biowest), and 1 mM sodium pyruvate at 37°C in a 95% humidified air-5% CO_2_ incubator. Cell passages were carried at 80% confluence at a ratio of 1:3, using trypsin EDTA (Sigma) to detach cells.

HT-29 cells were seeded at 2 × 10^4^ cells/well in 100 μl medium in 96 wells microplates (BD Falcon). Cells were allowed to attach and grow for 24 h under 37°C, 95% humidified air-5% CO_2_ incubation conditions before treatment with Fas ligand samples. Serially diluted Fas ligand samples (0.1, 1, 10, 100 and 1000 ng/ml) mixed with or without anti-FLAG® M2 antibody (Sigma, 2 μg/ml) were incubated for 48 h or 72 h in a final volume of 100 μl medium. The control experiments and the vehicle experiments consisted of medium only treatment and sample dilution buffer (50 mM sodium acetate plus 150 mM NaCl, pH 5.6) in medium treatment, respectively. Either of them was incubated with or without 2 μg/ml anti-FLAG® M2 antibody.

Cell viability was evaluated using MTT assay. Ten μl of MTT (5 mg/ml in PBS) was added to each well at the end of the treatment, and the plates were protected from light and incubated overnight at 37°C in a 95% humidified air-5% CO_2_ incubator. Then, 100 μl of 10% SDS solution was added to dissolve the formed formazan, and the plates were incubated for extra 24 h under the same conditions. Absorbance at 570 nm was recorded using Power Scan plate reader (Dainippon pharmaceuticals). Cell viability was calculated as % of control. A series of experiments were conducted in triplicate for each independent condition. Duplicate series of experimental data (n = 6 in total) were used for statistical evaluation of cell viability.

## Abbreviations

hFasLECD: Human Fas ligand extracellular domain; hFasRECD: Human Fas receptor extracellular domain; hFasRECD-T-Fc: A fusion protein composed of human Fas receptor extracellular domain and human IgG_1_-Fc domain containing a thrombin cleavage site within the fusion-region sequence; aa: Amino acid residues; BMGY medium: Buffered glycerol complex medium; BMMY medium: Buffered methanol complex medium; TCEP: Tris-(2-carboxyethyl)phosphine; EDTA Na: Ethylenediaminetetraaceticacid sodium salt; PEG: Polyethylene glycol; NaCl: Sodium chloride; SDS-PAGE: Sodium dodecyl sulfate polyacrylamide gel-electrophoresis.

## Competing interests

The author has applied to the Japan Patent Office for a patent relating to the content of this paper.
